# ‘We don’t talk about that around here’: an interpretative phenomenological analysis (IPA) of South Asian male survivors’ experiences of childhood sexual abuse in the UK

**DOI:** 10.1186/s40359-025-02706-z

**Published:** 2025-08-13

**Authors:** B. Kennath Widanaralalage, Stacey Jennings, Coral J. Dando, Jay-Marie Mackenzie

**Affiliations:** 1 Department of Psychology, King’s College London, Guy’s Campus, Great Maze Pond, London, SE1 1UL UK; 2 School of Social Sciences, University of Westminster, London, UK

## Abstract

**Background:**

Sexual violence against men is an understudied issue, particularly among ethnic minority groups. This study explored how South Asian cultural norms shape disclosure and help seeking for adult male survivors of childhood sexual abuse in the UK.

**Methods:**

Using interpretative phenomenological analysis (IPA), semi-structured interviews were conducted with 11 South Asian male survivors of childhood sexual violence currently living in the UK. Participants were recruited through specialist support organisations. Interviews explored participants’ experiences of disclosure, cultural factors surrounding sexual violence, and barriers/facilitators to support. Data were analysed following IPA’s idiographic approach to identify themes across participant accounts.

**Results:**

Three key themes were identified centred on familial honour and pressures, cultural taboos, and barriers to support/justice. Familial reputational pressures made survivors reluctant to disclose and they often received dismissive reactions. Cultural taboos about sex and mental health meant survivors felt unable to recognise their experiences and needs. Barriers to professional support included stigma and lack of cultural understanding. Distrust of systems discouraged justice-seeking, with negative experiences for those reporting abuse.

**Conclusions:**

Traditional South Asian values regarding family honour, gender norms, and stigma created obstacles to disclosure and help seeking for male survivors. Culturally-tailored outreach and supports are needed to overcome taboos and empower South Asian male survivors to access appropriate care. Findings emphasise the intersection of masculinity and culture in shaping experiences of sexual violence. Further research should explore diversity within South Asian communities and experiences navigating systems and services.

**Supplementary Information:**

The online version contains supplementary material available at 10.1186/s40359-025-02706-z.

The issue of sexual violence against men and boys is gaining increasing academic attention in the United Kingdom (UK). Researchers have highlighted how men navigate and make sense of their experiences of sexual violence [1, 2] amid substantial social and institutional barriers to accessing mental health and criminal justice services [3–5]. A growing body of research on violence against men and boys is emerging from different global contexts [6–10], challenging the traditional perception of sexual violence as exclusively a ‘female issue’ [11]. Findings from these studies underscore that male sexual victimisation is a cross-border phenomenon affecting individuals from diverse backgrounds, sexual orientation, ethnicities, cultures, and age groups. Although prevalence estimates vary, research from different cultural settings emphasizes the importance of understanding how cultural identities shape the lived experiences of male survivors of sexual violence, influencing their sense-making, recognition, disclosure, and help seeking behaviours [12].

The cultural and ethnic diversity of the UK population demands a thorough examination of how sexual and intimate partner violence, particularly child sexual abuse (CSA), might manifest in previously unexplored or overlooked settings. Indeed, recent studies have highlighted the prevalence of such violence and victimisation among ethnic minority women [13, 14] and men [15, 16] and how cultural identities and values of minoritised ethnicities in Western society may exacerbate well-known challenges for male survivors of rape and sexual abuse, particularly regarding rigid masculine norms and perceptions of male invulnerability [2]. Therefore, the present study aims to explore how cultural norms and values shape the experiences of adult South Asian male survivors of rape and CSA in the UK. Specifically, the research seeks to understand the barriers to disclosure and help seeking and examine their experiences accessing professional support and criminal justice services.

## Background

Global estimates suggest that between 5 and 25% of men and boys experience sexual violence at some point in their lives– from childhood through to adulthood [17]. This broad range in statistics results from differing definitions of sexual violence, varied measurement tools, and diverse research methodologies employed to study this complex issue. Notably, certain demographics are disproportionately affected; for instance, research shows that men aged 18 to 24 attending university are five times more likely to face sexual assault compared to their peers who are not in higher education [18]. Furthermore, men identifying as gay, bisexual, or those who engage in sexual activities with other men are frequently at higher risk [16, 19, 20]. Particularly striking is the finding from UK police data that reports higher rates of CSA amongst males under 14 compared to females [21]. Despite these established prevalence rates, it is crucial to recognise that significant underreporting, particularly among male survivors of CSA, continues to obscure the true scale of the issue [22, 23]. This pervasive silence is driven by stigma, societal perceptions of masculinity, and systemic barriers to disclosure, all of which contribute to the challenges in accurately capturing the extent of male sexual victimisation.

The mental health consequences of male sexual victimisation are profound and multifaceted, often exacerbated by societal stigma, internalised shame, and inadequate support services [24]. Cultural narratives that frame sexual violence as a women’s issue marginalise male survivors, fostering isolation, self-blame, and reluctance to acknowledge their victimisation. This stigma reinforces doubts about masculinity, fears of being judged based on sexual orientation, and anxiety over societal perceptions [25–27], further compounding psychological distress. As a result, many survivors experience PTSD-related symptoms, anxiety, and depression, often rooted in feelings of powerlessness, guilt, and worthlessness [28–30]. The lack of validation and recognition of their trauma further intensifies these issues, as survivors frequently feel their experiences are minimised or dismissed by society, professionals, or even close acquaintances [2]. The cumulative impact of stigma, shame, and inadequate support contributes to high rates of suicidal ideation and self-harm [31]. Moreover, the limited availability of gender-sensitive mental health interventions, coupled with the risk of disbelief or insensitivity from professionals, deters survivors from seeking help, perpetuating a cycle of untreated psychological harm [2, 32]. Fears of judgment, blame, or rejection often prevent disclosure, delaying recovery and obscuring the true scale of sexual violence against men. These barriers to disclosure and support may be further exacerbated in communities where cultural expectations of honour, family reputation, and rigid gender norms heighten the fear of stigma, ostracisation, or bringing shame to one’s family.

### South Asian male victims of sexual violence

South Asian ethnic groups make up over 8.5% of the population in the UK. Collectively, they are the largest and fastest growing ethnic minority group in the UK. However, there remains a noticeable gap in research and understanding regarding the experiences of minoritized men from South Asian ethnic groups, especially in the context of male sexual victimization and the intersection of gender, sexuality, ethnicity, and culture. Indeed, limited and mixed evidence exists regarding the prevalence of sexual violence amongst ethnic minority men in the UK in general, with rates ranging between 5 and 6% [24, 25]. Some evidence suggests that as many as 27% of ethnic minority men who have sex with men in the UK have experienced sexual abuse in their lifetime [16]. These figures are likely underestimates due to the pressures associated with concealing male sexual victimisation per se, which are often exacerbated for survivors from ethnic minority groups. Beyond a socio-cultural stigma around male sexual victimisation, taboos and secrecy around one’s sexual activities and orientation within South Asian communities can create additional barriers for disclosure, with men fearing losing familial and social support [26].

*Izzat* (honour) and *sharam* (shame) are core social constructs deeply embedded in South Asian communities, significantly influencing social behaviours, interactions, and mental health [27]. These constructs often serve as mechanisms of social control, affecting both men and women in their help seeking behaviours [28–30]. Izzat governs personal and family reputation, closely tied to social standing. It influences decisions on personal matters, such as reluctance to seek support outside the family and community. In South Asian communities, maintaining family dignity and respect is emphasised, often discouraging actions perceived as dishonourable or exposing vulnerabilities. These expectations can be particularly burdensome on boys and adolescents, who are seen as future providers and bearers of family honour [31]. Traditional gender roles may exacerbate these pressures, as young males might be expected to demonstrate strength, control, and success in their personal and professional lives [15]. Relatedly, sharam acts as a powerful deterrent against seeking help, especially for sensitive issues like sexual violence and abuse [30]. The fear of shame is not just personal but extends to one’s family and community, perpetuating silence around issues that might be perceived as dishonourable. In South Asian societal norms, where collectivism and interdependence are paramount, individual actions are seen as reflections of the entire family or community [32].

As survivors of sexual violence often report poor mental health, highlighting their increased need for mental health support [33], cultural contexts can create additional barriers for South Asian survivors, whereby seeking support for mental health issues may be perceived as a sign of weakness or a burden on the community. Indeed, evidence suggest that several socio-cultural barriers contribute to the underreporting of mental health concerns in South Asian countries and communities [34]. Men, traditionally seen as the family’s pillar, may resist acknowledging vulnerabilities to avoid tarnishing their izzat. Expectations of masculinity and the traditional role of men as providers and protectors have been found to contribute to the reluctance to admit vulnerability or seek help for mental health concerns [35]. Taken together, the evidence highlights how mental health stigma and barriers are deeply intertwined with the broader socio-cultural pressures faced by male survivors of sexual violence—pressures that inevitably extend to children as they navigate similar societal expectations and constraints.

Recent work by Gill and Begum [15] supports this notion by providing insights into the experiences of British South Asian male survivors, shedding light on disclosure and cultural norms. South Asian male survivors in their study opted for disclosing to non-family members, for fear of ‘letting down’ the family and compromising the family standing in the community. Some survivors in the study expressed doubt over whether family members would believe them, thus pushing survivors to protect themselves from negative reaction to their disclosure. However, little is known about the subsequent stages of survivor lived experiences regarding help seeking and reporting, emphasizing the necessity for further research. For instance, cultural influences play a significant role in shaping how female survivors from South Asian communities interpret their trauma responses, involving fears of dishonouring the family. These cultural factors impact on how individuals navigate and disclose their experiences [28–30, 36, 37]. Conversely, concerns about dishonouring and shaming often lead families to cover up incidents of sexual violence against female members, fearing that it may tarnish their status in the community [29, 30]. Therefore, it is reasonable to question whether similar familial pressures influence South Asian men and boys’ help seeking behaviours following sexual abuse.

### The current study

Despite the growing academic and policy focus on sexual violence experienced by men and boys, there is a critical gap in research focusing on minoritised groups, particularly South Asian communities. The existing literature strongly underscores the need to comprehend the intersection of masculinity, culture, and migration in shaping the experiences of disclosure, help seeking, and recovery for South Asian male survivors of sexual abuse. Notably, there is a lack of research on survivor encounters with formal help seeking processes and reporting to criminal justice services. The current study seeks to amplify the voices of this doubly disenfranchised group, situated at the crossroads of gender and ethnic marginalization, by presenting the narratives of South Asian male survivors of childhood sexual violence in the UK. Here we qualitatively explore survivor lived experiences of sexual violence through a phenomenological inquiry, delving into how they navigate disclosure, the cultural factors influencing their experiences, and the barriers and facilitators for accessing professional mental health support and reporting to the police.

## Methods

### Participants

In line with recommendations for IPA studies, purposive sampling was adopted to locate a defined group for whom the research problem has relevance and personal significance. Purposive sampling aligns with the theoretical underpinnings of IPA, designed to achieve a broadly homogenous sample and place focus on common patterns of experiences, whilst examining the uniqueness of each individual account [38]. As such, IPA typically favours smaller samples (*N* < 12) to yield the level of in-depth examination required to produce experiential accounts [39]. The target population in this study consisted of South Asian male survivors who experienced sexual violence. The terminology adopted when referring to these ethnic minority groups reflected current national guidance whereby the term Asian refers to Bangladeshi, Pakistani, Indian, and Sri Lankan heritage. In line with current recommendations, participants were asked to self-ascribe their ethnic group. As this study was concerned with the UK context, eligible participants included those who had experienced sexual violence or attempted to disclose or access support in the UK (including reporting to the police).

Mindful of the ‘hidden’ nature and enhanced stigma encountered by this group [17], the research team adopted a multiphase approach to recruitment. Firstly, the team conducted a mapping exercise to identify relevant UK based support services by undertaking an expansive search of both national and local providers. Specialist organisations for male sexual violence were contacted initially, followed by non-gender-specific sexual and domestic violence organisations, and lastly affiliated organisations that offer support to specific relevant subgroups (e.g., South Asian organisations, LGBTQ+) or for linked issues (mental health and addiction support). Regional hubs of national organisations were contacted individually as the demographic makeup of certain locations were more likely to reflect the target group under study. Most organisations advertised the study to clients within their networks and directed those interested to contact the research team. Some services also circulated the study flyer on their social media platforms. A member of the research team then contacted the participant to discuss the study, confirm eligibility and schedule a research interview at the participant’s convenience. A total of 11 male survivors were recruited to take part in one-to-one, semi-structured online interviews. Table 1 outlines participants’ demographic and case-related information.

**Table 1:** Demographic and case-related information

	*N* = 11
**Age**	
20–30	3
30–40	5
**>** 40	3
**Ethnicity**	
Bangladeshi	1
Pakistan	5
Indian	3
Asian: Other	2
**Sexual Orientation**	
Heterosexual/Straight	10
Preferred not to say/Other	1
**Incident Type**	
Rape	1
Assault by Penetration	1
Sexual Assault	3
Forced to penetrate	1
Sexual Abuse	5
**Age at the time of the first incident**	
3–5	3
5–10	6
10–16	2
**Accessed support**	
Yes	7
**Reported to the police**	
Yes	8

### Materials

Participants took part in one-to-one semi-structured interviews. An interview schedule was developed based on existing literature and previously identified critical research gaps (see supplementary files). This schedule was used flexibly to allow for the natural flow of conversation between interviewer and participant. The interviews thus took the form of largely open discussions on participants’ experiences of male sexual violence. The key areas covered in the interview included: (a) their experience of sexual violence, (b) ethnic, socio-cultural, and religious factors surrounding male sexual violence, (c) barriers and facilitators around disclosing their victimization, and (d) the barriers and facilitators to access effective therapeutic care and reporting to the police.

### Process

Upon recruitment, all participants received digital copies of an informed consent form detailing the general purposes of the study, to be completed before the arranged video interview. This form emphasized their rights in terms of confidentiality, anonymity, and withdrawal. Given the sensitivity of the topic under study, the research team offered flexibility to participants preference in method of interview (online or in person) as well as interviewer characteristics (male or female). All interviews were remotely conducted and securely recorded on the Microsoft Teams video conferencing platform. The interviews lasted on average 1 h (ranging from 30 min to two-hours). During the interview, close attention was paid to participants’ well-being and potential signs of distress. Participants were made aware they could take breaks and were encouraged to do so when needed. Provision of relevant professional support resources were also made available ahead of interview.

Post interview, participants were invited to a debrief session where the aims and objectives of the research were reiterated. The interviewer also reminded participants about support services available and ensured that they had access to the relevant contacts. A copy of the debrief form was sent to participants detailing this information, as well as the authors’ contact details so that participants could get in touch to share additional information or reflections on their experiences. Participants were offered a £20 voucher as a thank you for their time. All audio recordings were immediately uploaded to secure password-protected servers post and recordings deleted once transcribed. To preserve anonymity, participant names were replaced with appropriate pseudonyms. Any additional information provided in analysis excerpts that could identify an individual were also anonymised.

### IPA

IPA was deemed the most appropriate method of analysis given the aim of understanding the participants interpretation of their experience of sexual violence. The theoretical underpinning of IPA stems from a phenomenological stance of double hermeneutics, concerned with the researcher’s interpretation of the participants understanding of a particular phenomenon [39]. IPA is also idiographic, with an analytic focus on appreciating the uniqueness of the singular experience, before constructing broader trends or “group experiential themes” in the overall sample [39]. As such, IPA is suitable to access hidden voices from seldom-heard groups by “metaphorically shining a light” [40]. The researchers endeavoured to transparently demonstrate how the established philosophical principles characteristic of IPA have been followed in the analysis [39]. The interviews were transcribed verbatim to stay as close to the participants’ natural conversations as possible, including pauses and hesitations [41].

The analysis followed the stages of IPA described by Smith et al. [42]: reading, exploratory notes, constructing experiential statements, seeking relationships and clustering into personal experiential themes, and grouping to identify group experiential themes. Each transcript was examined individually in line with the idiographic underpinnings of IPA. This process was then repeated for each of the interviews to generate an overall master table of Personal Experiential Themes (PETs). PETs across each transcript were then compared and contrasted to identify Group Experiential Themes (GETs) that permeated participants’ experiences. The first author’s own views and assumptions were regularly reviewed and discussed between the research team in a collaborative process; an integral part of the analysis process. All authors independently examined and refined the personal experiential themes and master themes identified for each account. All authors were involved in critically assessing the validity of the themes against the original participant transcripts to ensure that the extracts selected captured the range of male rape experiences within the sample of this study. All methodological and analytical decisions taken were in line with established validity and quality guidelines for qualitative and IPA research, further discussed below.

### Validity and quality assurances in this study

Enhancing attributes of rigour and credibility has been a longstanding area of priority within qualitative research [43]. As such, care was taken to ensure that this study was designed, conducted, and reported according to current best practice guidance for both qualitative research, and IPA specifically. In this study, validity was defined as the “extent to which the design and methodological approach used in a study are fit for purpose” (J. A. Smith et al., 2022, p. 147). Three guidelines were used to ensure that the methods employed were appropriate and coherent to the research questions: four principles for good qualitative research [44], Smith’s assessment tool for quality in IPA studies [45], and four quality indicators for IPA research [46]. By closely and carefully ensuring that the analysis reflected IPA’s philosophical principles and that the design of the research was transparent and rigorous, the authors engaged in a collaborative and iterative process to present vigorous accounts of how participants made sense of their experiences.

Examining ethnicity raises prominent ethical considerations that required due attention in this research. Whilst researchers in this field have a duty to reduce inequality, it is important to balance the benefits alongside the potential negative impact of stigmatizing and perpetuating stereotypes of ethnic groups. Accordingly, this study consulted the ethical principles for ethnic health research laid out by the Leeds Consensus Statement [47], in addition to updated governmental standards on ethnicity data in research [48]. These principles were reflected in framing the focus of the research, data collection and analysis. For example, we included defined ethnic minority subgroups (e.g., Pakistani, Indian, Bangladeshi) rather than utilising aggregate categorisation. We also paid due regard to how ethnicity intersects with other relevant factors including gender and religious identity to develop a sophisticated/fuller understanding of experiences in the sexual violence context. Participants were also invited to self-ascribe their own ethnic identity to ensure ethnic categorisation was meaningful in relation to the experiences and outcomes being explored.

## Results

**Fig. 1:**
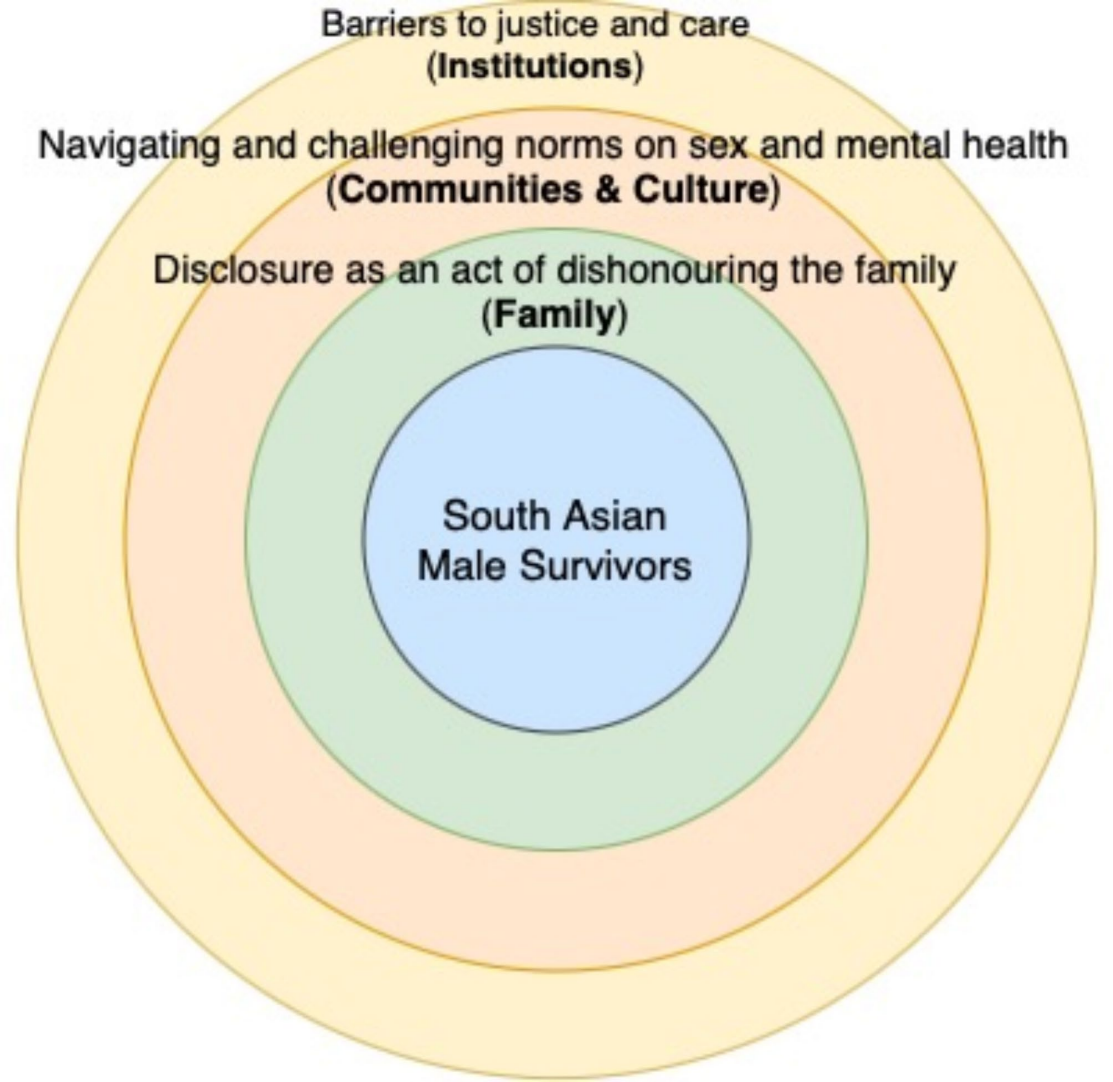
Thematic map of group experiential themes

South Asian men’s experiences of sexual violence were best captured by three group experiential themes which focused primarily on survivors’ childhood experiences around: [1] disclosure as an act of dishonouring the family; [2] navigating and challenging norms on sex and mental health; and [3] barriers to justice and care. This thematic representation underscored a complex web of interconnected barriers and challenges, wherein familial pressures, societal taboos, and systemic barriers converged to shape survivors’ childhood experiences of sexual abuse. While each theme represents a distinct layer of experience, they are not isolated; rather, they build upon and reinforce one another, forming a cumulative burden that survivors must navigate. The interplay between familial expectations, broader cultural norms, and institutional barriers illustrates how challenges are not experienced in isolation but rather in a layered and compounding manner, with each level shaping and intensifying the next (Fig. 1).

### Theme 1: It’s a matter of honour: disclosure as an act of dishonouring the family

Participants frequently described how the cultural emphasis on honour and family reputation resulted in internal conflicts and reluctance to disclose their experiences. When discussing their experiences and decisions around who to tell, participants often reflected on challenging family dynamics that negatively impacted their confidence in openly discussing their experiences of victimisation. As such, the idea of disclosing to their families was often thought of as an act that would bring shame to the family.

I’ve always felt from the wider family quite heavy expectations to be successful, to perform well academically and professionally. The expectations felt quite heavy [so] I don’t feel like opening up about what happened to me, even though I think it’s shaped me as to who I am today. It constricted me, the weight of familial expectations where you must be a certain type of man. And being someone who went through sexual trauma as a child doesn’t fit with that because it makes me feel like I’m not the type of man that I should be…you’re not meant to have those kinds of weaknesses or vulnerabilities, whatever, which are holding you back in certain areas of your life. (Ijaz)

In this extract, Ijaz’ poignantly highlighted the struggle between ‘opening up’ and the ‘weight’ of familial expectations, noting how the burden of being the bearer of the family honour intensified his feelings of vulnerability as he grappled with his experiences of CSA. Ijaz’s account highlighted how family and community pressures to achieve professional success influenced his reluctance to disclose his sexual abuse, as he felt he failed to meet the standard of a successful man. While Ijaz was aware of the irreconcilable conflict between family expectations and his own needs, his reluctance to disclose emphasised a common worry among survivors of family reactions to revelations of sexual violence. Survivors feared disclosure and described it as a last resort.

However, survivors’ reservations were confirmed as families reacted negatively and dismissively. For example, Charan described his family’s attempt to minimise his abuse, by questioning the credibility and significance of his account:

When these things happen, we are constantly told to brush it under the rug as it’s a matter of honour and the honour of the family name. It’s something that they don’t want to ruin, for society’s purpose. And I was constantly told that no one would believe me because I’m younger and my abuser was older and people generally believe the older people over the younger people, as a matter of respect… I felt like my family never took me seriously. They always took me for a kid who is being rebellious and not someone who was physically and mentally going through a traumatic experience (*Charan*).

Charan’s experiences of disclosing in adulthood showcased how South Asian male survivors often found themselves negotiating between their needs and vulnerabilities and familial pressures to conceal their experiences of abuse. As such, disclosures of abuse were seen as assaults to family’s standing within their community, creating an environment in which participants felt dismissed and rejected by their closest support network. Charan’s account of his CSA experiences resonated with many participants in this study, who were often treated as if they were intentionally challenging the family values. Importantly, comparing Ijaz and Charan’s reflections of disclosure, familial pressures were evident before, during, and after survivors’ first attempts to discuss their experiences with their loved ones– thus, highlighting the comprehensive impact these pressures have on survivors’ well-being and decision-making process regarding disclosure in childhood or in adulthood.

While the degree of rejection varied, survivors’ unanimously felt that shame was as important as honour to understand their families’ reaction:

They [*the family*] ask for forgiveness [*for the abuser*], you need to find a way to forgive and move on and live like one happy family. They just don’t understand that it is not possible. It is not a mistake. It’s shame the biggest issue that we have is - my mum says ‘If this comes out, and you want to make it official, what’s it going to achieve? What’s he going to do? What are people going to say?’… As soon as I went home, [the abuser] was there, went on his knees, started crying, begging for forgiveness. Mum just sits and just looks at me and says ‘He’s apologised. What, what are you going to get from speaking up about it now? You need to move on.’ (*Ravi)*.

Ravi’s testimony highlights the profound feelings of isolation and alienation that male survivors of CSA often experience. This sense of estrangement is particularly evident in Ravi’s recounting of his mother’s response - rather than receiving support, he was met with an expectation to forgive the abuser, a close family member, and to ‘move on’. Such responses trivialise the survivor’s trauma, relegating it to a ‘mistake’ that must be concealed to protect the family’s honour and avoid community disgrace. It underscores the complex challenges faced by survivors whose abuse happens within the family. In Ravi’s case, key family members, such as his mother, are pivotal in perpetuating a cycle of shame. By dismissing disclosures as distasteful and unnecessary, they inhibit the healing process and reinforce the survivor’s isolation. Disclosing abuse is a crucial step for survivors of all genders, as it is often made to individuals they trust and share intimacy with [33, 42, 49]. However, when such disclosures are met with dismissiveness or negativity, as in Ravi’s experience, it further alienates the survivor. The need for recognition, understanding, empathy, and support is central to the healing journey of CSA survivors. Yet, as illustrated by Ravi and others in this sample, societal and familial reactions frequently fall short, leaving male CSA survivors feeling even more isolated.

Conversely, some survivors reported how familial CSA created pressures and conflict between different factions of the family. Amir found himself at the centre of arguments between family members seeking ‘revenge’ or wanting to fully conceal the incident:

I was feeling really bad. I was thinking ‘What am I doing? I’m breaking the family up.’. I [felt I was] breaking the family up because obviously I’ve got emotional ties to me mother, sister, but also to [the abuser] as well. It’s really hard… So, all my uncles and aunties and stuff from that side of the family were saying, ‘Let us come up, we’ll talk and we’ll deal with it. Forget about going to the police’. They started having problems and arguments with each other, my mother with [the abuser’s] side of the family. Which exist to this day, unfortunately. *(Amir)*

Amir’s account vividly illustrates how the family’s deep-rooted emphasis on honour dictated their response to his disclosure. Each side was preoccupied with preserving their social standing, turning his revelation into a source of tension and conflict rather than support. The distress this caused left Amir deeply regretting his decision to share his experiences, as he became the unwilling catalyst for family disputes. His narrative underscores the profound guilt that often accompanies disclosure, as survivors find themselves burdened not only by their trauma but also by the repercussions their truth has on family dynamics. Rather than receiving validation and support, Amir’s disclosure exacerbated divisions within the extended family, overshadowing his own need for healing. His experience highlights the complex web of familial expectations, where maintaining honour takes precedence over a survivor’s well-being and protection. These pressures profoundly shaped how survivors perceived their disclosures—what began as an act of self-realisation and self-actualisation quickly became an obligation to manage the family’s concerns about reputation and social standing.

### Theme 2: navigating and challenging norms on sex and mental health

Participants’ accounts revealed how cultural taboos and restrictions surrounding sexuality in South Asian communities profoundly impacted their experiences as survivors. As honour and shame are fundamental social constructs in South Asian communities [50], the accounts gathered in this study reflected how sexual violence was frequently juxtaposed against broader cultural taboos and restrictions related to sex and sexuality. These taboos significantly shaped survivors’ experiences, serving as the backdrop for their feelings of guilt and shame for their CSA experiences - often prompting them to suppress sexual thoughts. For instance, Ijaz recalled how his family’s religious beliefs made his CSA experiences even more distressing and uncomfortable:

I grew up in a Muslim household… sex was never really spoken about, and the expectation was always no sex before marriage, you shouldn’t have any girlfriends, no masturbation. I always associated sexual feelings with a certain amount of shame… And I’d always try and suppress any kind of sexual feelings that I had growing up…growing up I never even considered the idea that I could have been vulnerable to sexual victimization or anything like that…so if I had my own questions about it or things that were on my mind, I would never really open up about those to either of my parents or you know, ask about it, because it was something which was in a taboo area. (*Ijaz*)

Having been taught to associate sexual thoughts with feelings of shame, Ijaz later in the interview revealed that he kept his CSA experiences with a female family acquaintance a secret for over 15 years. The culture of shame surrounding sex, as described by Ijaz, can act as a barrier to recognising, disclosing, and coping with CSA experiences. Survivors often fear that their experiences may challenge cultural norms and beliefs, leading to a reluctance to come forward. Ijaz’s account underscores the struggle many survivors face in reconciling cultural norms that stigmatise sex with their own unwanted sexual experiences, particularly those involving trauma at a young age. The cultural taboos surrounding sexuality create barriers to disclosure, which are further compounded by internal feelings of shame and self-blame. These factors significantly impacted Ijaz’s ability to even recognise, let alone disclose, his experiences of sexual violence.

In addition to dealing with taboos about sex, participants often had to overcome negative attitudes towards mental health. Participants described how their mental health concerns were often misunderstood or dismissed by their families, who seemed incapable of understanding the gravity of their CSA experiences. These reactions were often intensified by survivors’ delayed disclosures, as families struggled to understand why and how events from many years ago continued to affect them:

I’ve suffered a lot from mental health issues and personally, I felt like my family never took me seriously. They always took me for a kid who’s being rebellious and not someone who was physically and mentally going through a traumatic experience. And I feel like without that education my parents were just taking me for a joke… I just didn’t feel like I received any support, and even today, like I still suffer for mental health, but my parents just constantly tell me to get over it. As if it’s a feeling that I can get over and not a mental illness. for the first few months they were understanding, but then after that they were just like get over it- it’s happened, the police know, so just get over it. As if it’s not a valid feeling for me. (*Charan*)

Charan was one of the few survivors who attempted to openly discuss and describe his mental health struggles after the abuse, in adulthood, but was met with unsympathetic responses. His account resonated with others in this study, illustrating how CSA experiences are seen as challenges to socio-cultural norms around masculine resilience and invulnerability from what are seen as ‘normal’ life struggles. Familial pressures around success and honour meant Charan’s mental health needs were seen as secondary. Hostile cultural norms that stigmatise mental illness compounded the issue, creating an environment where Charan felt unable to recover on his own terms. His experience reflects how family dynamics in South Asian communities often dominate and rigidly police norms for their members. Charan felt constrained by these traditional standards that discouraged open discussion of mental health struggles. Overall, the lack of awareness around abuse and prioritization of masculine ideals over Charan’s wellbeing prevented him from getting the sensitive support he needed from his family after trauma.

Discussions of abuse and mental health needs were often seen as intentional attempts to challenge the culture and norms of the community. For example, Rowan explained how being open about his abuse was seen as violating norms around gender and sex, with the community viewing such discussions as embarrassing:

If you do mention it, they’re going to look at you down. They’re going to think you’re odd. You’re weird, you’re queer or you’re gay. And obviously these all things are not very… even though as a society we’ve changed and attitudes have relaxed, but especially in the Muslim and Asian community, it’s going to be really embarrassing to bring up something quite personal like that. (*Rowan*)

Rowan’s disclosure of his abuse in adulthood within the community was largely met with negative and sometimes homophobic reactions, reflecting male rape myths [33]. This highlights the perception in South Asian contexts that male survivors challenge traditional gender and sexual norms, which are often considered private matters not to be openly discussed, especially when the abuse involves a crime committed within the community. The dismissive and embarrassed responses to Rowan’s *perceived* sexual orientation illustrate how cultural taboos surrounding sex, gender, and mental health impede survivors from openly sharing their experiences, leading to detrimental effects on their mental wellbeing.

The role of culture in shaping survivors’ post abuse experiences became especially apparent when participants directly compared South Asian and White British values.

Talking about sex in the Indian culture is taboo in general. In Bollywood films for example kissing on screen has only started to happen in the last 15–20 years and even still it is considered very distasteful, and those films are considered dirty and cheap, as the culture is all about respect and family. I have never disclosed this to people because it has been too embarrassing and difficult to talk about without judgement and fear of it negatively affecting my mental health. I have never disclosed it to people of my Indian ethnicity up until very recently because the culture is very judgemental and the mentality appears to be very backwards and is not progressive, such as it is in the UK. (*Baira*)

In an Asian family, you’re going to struggle more to speak up about this, right? […] your English family is a lot more open and receptive to this kind of thing. Whereas in Asian community, if you’re a child and you’re trying to speak out, you’re going to get silenced, quickly. You know, your mum and your dad are going to tell you ‘We don’t talk about that around here. Don’t worry about it. You’ll get over it.’ (*Jaz*).

Baira and Jaz’s accounts reflect the experience of surviving abuse as ethnic minorities, navigating and observing different cultural values from their families and the surrounding community, which provide various lenses for understanding and rationalizing experiences of CSA. Disclosure felt impossible for them, as fears of judgment and impacts on mental health were compounded by the awareness that their families and South Asian community seemed unequipped or unwilling to provide appropriate, sensitive care. Many survivors observed the disparity in awareness and support provided to victims from other ethnic background (i.e. White), thus emphasising feelings of constraint from traditional South Asian values and norms. The disconnect between their heritage and the resources available to them as CSA survivors exacerbated fears of judgment and mental health impacts, making disclosure seem impossible.

### Theme 3: ‘going undercover’: barriers to justice and care

Despite the challenges and pressures experienced around recognition and disclosure, many in this study attempted to report to the police and seek mental health support after their abuse. However, traditional cultural values and attitudes within their communities, as well as issues with services in criminal and healthcare systems prevented and deterred participants from reporting their abuse and accessing care. Survivors described feelings of shame, taboos around topics like sexual abuse and mental health, dismissal or discrimination from authorities, and a lack of culturally-competent resources as key factors that compounded their trauma. Their experiences reveal how social stigma, tight-knit family structures, the marginalization of minority groups, and distrust of systems can make seeking support or reporting abuse feel like an act of defiance—one that forces survivors to go under cover, navigating secrecy and fear to avoid shame or community backlash. This section will explore the specific obstacles South Asian male survivors encountered when navigating disclosure, community responses, mental healthcare, and the criminal justice system across two subthemes: *reporting childhood sexual abuse* and *accessing support*.

### Reporting childhood sexual abuse

When asked about reporting to the police, Ijaz presented his rationale for not involving the police whilst acknowledging feeling somewhat unsettled by the lack of closure for not seeking justice:

It’s not even an option for me, it happened so long ago… It’s just one of those things where I just know there’s not going to be any justice for it and I do worry because I don’t know how many other boys might have hurt and maybe [the abuser] is still hurting, but it’s not something which I can, you know, like it wouldn’t lead anywhere if I were to report it to the police or anything like that. And I think that’s another thing, there’s just no closure from it, or no healing really. It’s just one of those things where it’s just going to be continuously kind of open-ended because there’s not going to be any justice at the end of it, you know? (*Ijaz*)

Ijaz’s assessment of his situation and his decision to not report revealed an internal conflict between the reality of taking part in a lengthy criminal investigation and the moral duty of reporting a perpetrator who could still be abusing others. This highlights a common challenge for male survivors of historic CSA [2] who feel disempowered to seek justice because it could jeopardize their recovery. This risk became reality for Charan and others in this study who did report to police. In hindsight, Charan felt he would not have reported if he knew what he would go through, emphasizing the need for trauma-informed and empowering interactions with the criminal justice system:

It’s been a lot more negative than positive in my experience because it’s been months I’ve been waiting for a response for emails and phone calls, and then when I get a response it’s just very blunt. And then when I asked for information, I’m told that [*the police officer*] can’t provide that information to me. The only thing that’s come out of it that’s a positive was the Victim Support and getting help from Victim Support and then eventually to [specialist support service]. But with the police… I’ve not been able to concentrate on my personal life. Because [the case] is always been there, lingering in the background. It just makes me regret going to the police in the first place If I knew that this was going to happen, I would just forget about it and just get over it. (*Charan*)

Charan’s reporting occurred in adulthood, and his experiences with the Criminal Justice System were characterised by an intense frustration with officers’ poor communication during the investigation, both in terms of the quality and quantity of the information provided as well as the lack of sensitivity in their responses. Importantly, Charan’s account does emphasise how entering the criminal justice system often represents a gateway for accessing some support- in Charan’s case finding the specialist support he needed. Nevertheless, the feelings of regret permeated Charan’s account of his reporting experience, one where he felt it was more detrimental than beneficial for his mental wellbeing. Consistent with other accounts, Sunny’s experience of reporting left him wondering whether he could have been protected from further violence and trauma if police officers took his allegations more seriously:

I could have been saved in 2016 but the police failed me again. Where they were at the house, I had literally told them everything to raise a concern. Just imagine you’re a police officer, you’re at the house, I’m telling you I was physically assaulted, there’s a sexual abuse case going on against my [*the abusers*], I’m being emotionally abused throughout the entire investigation, I’m being psychologically abused, and then you basically just tell me to change my clothes and go for a walk and to cool down. But you don’t arrest [*the abusers*], you don’t do nothing, and they get away with everything. (*Sunny*)

[…]

When I look at what has happened, it’s that severe where the police just don’t even care about justice anymore. It just doesn’t even matter to them. And for me, I’d never go to the police again. Because right now, when you look at the police procedure it works completely against the survivor. (*Sunny*)

Despite providing substantial evidence of abuse as an adolescent male survivor, Sunny felt severely failed by the police in their response and follow-up actions. Sunny’s experience highlights how survivors seek validation and support from law enforcement. Like other accounts of negative reactions to disclosure, the dismissing and ineffective institutional response left Sunny disillusioned with the officers’ competence and ability to deliver deserved justice. As with other survivors in this study, Sunny questioned whether the police were truly serving abuse victims or if there was room to improve how cases in South Asian communities are handled. Critically, Sunny felt that a more thorough and sensitive initial response from the police could have likely prevented years of continued financial, domestic, and sexual abuse that followed in adolescence and adulthood. His account emphasizes the need for careful, trauma-informed handling of abuse cases and a justice system that survivors can trust will protect them and not add on to their trauma.

### Accessing support

Seeking professional help and support posed unique challenges for South Asian male survivors due to stigma surrounding both mental health and sexual abuse within their communities. To explain South Asian survivors’ hesitation in seeking professional support, Sal described how religious values and the misconception that sexual victimization only happens to sexual and gender minorities are cause for reluctance:

As Muslims we’re very, very keen on nuclear family, like, having a mum, dad, like any Abrahamic religion- it’s not the most LGBT friendly. So, a lot of lads that may have been abused don’t always go for help because they see that the help they might be getting is maybe from somebody…LGBT… Asian lads, the likelihood is they won’t talk about it. They won’t talk about it until they find the right person to talk to…So for example, I don’t think I could go to a Sri Lankan person and tell him my story because I don’t have any links with him. I know he’s Asian but he’s different. He’s got a different culture, a different religion, different… you know even to a Pakistani person. It’s not actually for me, it’s not an Asian thing. It’s a person that you could trust. (*Sal*)

The combination of stigmatising and hostile attitudes towards both mental health and sexual minorities highlights how debilitating such narratives can be for survivors seeking the appropriate support. As with disclosure, South Asian survivors must balance familial pressures, cultural value, and own mental health wellbeing. However, Sal also emphasised the importance of acknowledging the variability within different South Asian communities and how he felt he would not be able to confide with someone who did not closely share his cultural values and traditions. Trust and cultural identification are described here as key in Sal’s feelings around what felt as the appropriate support service for him. Charan further illustrated the struggle between these mental health needs and cultural dynamics, as he sought support in adulthood:

To be honest, it felt like [support] didn’t exist because I used to have my sessions with my therapist and then when I would go home there would be nothing on that topic and it would basically be like me going undercover to go have therapy and then coming back and trying to fake my emotions for the sake of my family. It’s honestly hard and it’s exhausting when you’re constantly having to fake a smile and inside, you’re just broken. It’s just hard to cope and most times I just like being alone because it’s exhausting to fake my emotions. So, if I’m alone, I can just be who I am. (*Charan*)

Associating going to therapy as ‘going undercover’ emphasises the taboos around mental health and the burden on South Asian survivors to conceal their emotional state to avoid the familial pressures that shaped survivors’ lived experiences of sexual abuse. Overwhelmingly, survivors’ shared Charan’s feelings of loneliness and fatigue from having to conceal their on-going journey from loved ones.

Although they often had to conceal their experiences and perceived a lack of cultural support, some participants were able to find help from specialist organizations that shared their values, later in life. Charan described the ongoing benefits of working with a counsellor of the same ethnic background:

I think that was really important because like, even though being in the UK, everyone has been brought under the same society, there’s still certain differences that carry on from culture to culture. And knowing that [service provider] shares the same kind of culture and upbringing as well as the British one, and the Pakistani one, that was something that was really helpful for me to know that he understands first-hand what an Asian household can be like. (*Charan*)

Finding support services with a shared cultural background was often critical for survivors’ willingness to access help and stay engaged in recovery. The dual identity of being both British and South Asian shaped how survivors made sense of their experiences. It allowed them to feel truly seen and understood when providers acknowledged this identity. Ravi, who was unable to find the culturally sensitive support he needed, felt that the lack of accessible services catering to Black Asian and Mixed Ethnic (BAME) communities caused reluctance among South Asian and other ethnic minority survivors in the UK to speak up. He believed tailored outreach and services were needed to help these groups open up about their experiences and get the right support:

One of the biggest issues we have about speaking up is what support is out there for people in the BAME community. The reason why I say BAME is that I know I come from a Pakistani community, but it’s not different, whether you’re Pakistani, Indian, Bangladeshi, Sri Lanka, Afro Caribbean, our communities are the same when it comes to stuff like this. And the reason why I say there’s not enough, nowhere near enough support in this country is because and I’ve said this at work as well, because I’ve tried counselling and I took some counselling sessions. A White counsellor is never going to understand the dynamics of BAME family. So, when I was having counselling in [*anonymised*], it’d be a common theme. Just cut off your parents, stop talking to them, walk away from your family. Our family dynamics don’t work like that. Even when we get married, we’re connected to the family, we live in together. And for me, it was trying to explain to my family why [*anonymised*] thinks the way he does, why does he have these beliefs? Why is he not able to live a normal life like you want him to? My family would never be able to understand that from a White counsellor. (*Ravi*)

In contrast to some other accounts, Ravi believed in the existence of shared experiences among ethnic minority groups in the UK. His experiences with support services emphasized that counsellors from white backgrounds may not always be equipped to deliver care that is sensitive or fully aware of the realities facing South Asian communities. Issues like family pressures and norms around sex and mental health are often not fully understood. Ravi and other survivors in this study pointed to examples like a service provider recommending breaking away from family as illustrating a lack of understanding of South Asian cultures, which have deep roots in family unity and tradition.

## Discussion

This research provides novel insights into the lived experiences of South Asian male survivors of sexual violence. All had experienced sexual abuse in childhood or adolescence, hence this study is centred on reflections as adult survivors, examining how they make sense of their experiences and navigate support systems in adulthood. This study does not investigate the prevalence of child sexual abuse within South Asian communities but rather explores how cultural and gendered expectations shape the long-term impact of abuse and survivors’ engagement with support services and the criminal justice system. Three group experiential themes were identified: disclosure as an act of dishonouring the family; navigating and challenging norms on sex and mental health; and barriers to justice and care. These themes illustrate how traditional values surrounding family honour, cultural taboos about sex and mental health, and systemic distrust can create significant obstacles to healing [15], highlighting the intersection of gender and culture in shaping survivors’ lived experiences. By centring narratives, we extend the limited research on this population, providing insights into how cultural influences shape recovery trajectories, disclosure processes, and engagement with support services and the criminal justice system.

Disclosure is a critical step in acknowledging sexual victimisation [49]. While challenges exist across survivor populations, differences emerge between child and adult disclosures, particularly regarding dependency and trust. Children are more likely to disclose abuse when supported by a validating caregiver [51], whereas those lacking support– as often the case in this study - delay disclosure until adulthood, navigating entrenched familial and cultural barriers. Participants showed how gender and culture significantly shape disclosure, particularly in South Asian communities, where men are expected to achieve personal and professional success as future family caretakers [52]. This pressure is amplified in immigrant families, where success is tied to family honour and social mobility [53, 54]. Fear of being perceived as a failure deters male survivors from disclosing, mirroring concerns among South Asian female survivors, for whom family honour and ostracisation are key barriers [55, 56]. However, gendered differences in scrutiny remain: while women are judged on purity and marriageability [57, 58], male survivors described fear around appearing weak or unmanly, reinforcing the intersection of masculinity norms and sexual violence stigma [12]. Self-evaluative emotions such as guilt, shame, and self-blame further deter disclosure, reducing access to care [2, 50]. Suppressing trauma is linked to poorer mental health outcomes [59], leaving South Asian male survivors particularly vulnerable. While disclosure can aid recovery when met with support, survivors frequently reported negative, dismissive, or hostile responses from family members. In many cases, family cohesion takes priority over individual wellbeing, particularly when perpetrators are relatives or respected community members [28], contributing to the silence and stigma surrounding sexual violence against men and boys in South Asian communities.

Survivors’ fear about their families’ reactions were deeply influence by cultural norms and biases surrounding sex and mental health. Traditional gender values are well-documented as shaping victim-blaming attitudes and rape myths for both female and male survivors [60, 61]. Asian communities are historically more conservative regarding sex and sexual violence than their non-Asian counterparts [54], and adhere to traditional values such as collectivism, humility, and filial piety. These values have been linked to internalised heterosexism and reluctance to disclose sexual orientation [62]. In line with this research, strict prohibitions on open discussions of sexuality meant many survivors in this study struggled to disclose their experiences and access support [36]. Shame and stigma are known barriers for male rape survivors, often preventing them from recognizing their victimisation [2, 50]. Additionally, cultural expectations of masculine resilience and stoicism [63, 64] were observed in this study, leading to the dismissal and stigmatisation of survivors’ mental health needs. Similar patterns are observed among South Asian women, where norms discouraging discussion about sex– especially in the context of violence– contribute to silencing and internalised shame [30]. Taken together, our findings highlight how gendered cultural expectations shape the experiences of CSA survivors, albeit through distinct pathways of stigma and social control.

Findings on professional support and seeking justice shed light on the challenges faced by South Asian male survivors which, to the authors knowledge, have yet to be explored. Similar barriers have been identified among South Asian female survivors, particularly their mistrust of formal institutions and reliance on informal, community-based support [65]. Women’s reluctance to engage with the justice system often stems from fears of disbelief, judgment, or further victimization [29, 30], and while male survivors share these concerns, their experiences are compounded by a lack of dedicated support services and cultural insensitivity within existing networks [5]. Questions and conflict around self-protections and duty towards potential future victims resonated with the accounts of many male rape survivors [2], emphasising the disempowerment felt by survivors in pursuing justice. While entering the criminal justice system often represents a gateway to specialist support [66], frustration and regret reported by participants reflected evidence of institutional betrayal trauma [67] often reported by service organisation who support men with lived experiences of abuse [50]. Stigma surrounding mental health in South Asian communities further restricted survivors’ access to professional support. Many struggled to navigate the tension between familial expectations, cultural norms, and their psychological well-being. The concept of ‘going undercover’ to access therapy highlights the additional burden imposed by societal taboos, forcing survivors to seek help discreetly while managing fears of exposure and judgment.

Thus, this study highlights the critical need for culturally sensitive mental health support for male survivors from ethnic minority groups. Cultural nuances, traditions, and values significantly shaped survivors’ experiences, with many reporting that culturally aware providers offered more effective care. However, a lack of cultural competence among service providers remains a major concern, limiting access to appropriate support. While recognising the diversity within ethnic minority communities, many survivors shared a sense of common identity and values associated with Asian communities, underscoring the importance of culturally competent services [68]. Such approaches could help address stigma, reduce barriers, and improve service engagement. Conversely, when providers failed to understand familial and cultural pressures, survivors reported feelings of stigma, distrust, and further trauma, reinforcing negative experiences of rape and CSA [69]. Some participants suggested ethnic matching between clients and providers, while others emphasised the need for professionals to have broader knowledge of Black, Asian, and Minority Ethnic (BAME) issues. However, ethnic matching is a complex approach, as seen in substance misuse literature for ethnic minorities [70]. A nuanced strategy integrating both ethnic matching and comprehensive cultural competence training could enhance support services, ensuring they are more accessible and effective for male survivors from diverse backgrounds.

### Implications for policy, practice, and research

Numerous implications for working with South Asian male survivors of sexual violence in the UK have emerged, centred on a need for greater awareness and sensitivity towards how ethnicity and culture shape how barriers emerge and are maintained. More research is needed to develop outreach interventions towards better understanding how to increase awareness whilst respecting the sensitive nature and pre-existing taboos around open conversations on ‘private matters’, such as sex and mental health. Recognising traditional values and fears of shame in these communities involves consulting community leaders and stakeholders to ensure that interventions are culturally sensitive and effective. A desire from South Asian men in this study for more visibility highlights the importance of platforms for discussion and political conversations towards delivering targeted and effective funding. A clear need for training and support for specialist organisation to increase their cultural competence in supporting South Asian male survivors towards culturally affinity and shared values in the client-provider therapeutic alliance [71].

### Limitations and future directions

Our sample size, while appropriate for a qualitative phenomenological inquiry, means findings may not generalise within South Asian communities. Participants were primarily of Pakistani and Indian descent, and so other ethnicities (e.g. Bangladeshi, Sri Lankan) are underrepresented. Reliance on self-selected volunteers also raises the possibility of selection bias. Notably, survivors who did not disclose their experiences and did not report to the police were underrepresented. Most survivors identified as heterosexual, and so the voice of sexual minority men from South Asian backgrounds were not captured in this study.

The overrepresentation of heterosexual participants in this study contrasts with existing research suggesting that men in same-sex relationships, particularly gay, bisexual, and other men who have sex with men, report higher rates of sexual victimisation [12]. Several possible explanations for this discrepancy warrant discussion. First, recruitment methods and the stigma surrounding both male sexual victimisation and LGBTQ + identities within South Asian communities are a likely contributing factor. Research indicates that Black and South Asian gay men in Britain face compounded barriers to disclosure due to fear of reinforcing negative stereotypes about their sexual orientation or experiencing additional stigma from their own communities [72]. Social and familial pressures within South Asian communities may also discourage LGBTQ + men from engaging in discussions about sexual violence, further reducing their visibility in research. Given deeply entrenched heteronormative values in many South Asian cultures, heterosexual men may have found it relatively easier to come forward, as their experiences do not challenge dominant cultural narratives in the same way LGBTQ + victimisation might. Future studies should consider targeted recruitment strategies to engage a broader spectrum of sexual minority survivors to ensure their experiences are adequately captured.

Finally, most participants experienced sexual abuse between the ages of 3 to 16, consequently the voices of adult males who experienced violence after the age of 16 is critically underrepresented, and more broadly in the literature [2]. It is evident how norms attached to male resilience, independence and strength [73] may be further enhanced for adult males from ethnic minority backgrounds as evidenced by survivors in this study who still displayed substantial levels of shame and embarrassment around disclosing their childhood and adolescent sexual abuse. Despite attempts to recruit men with adult experiences, the invisibility of male sexual violence within South Asian and other minoritized communities meant that this is an important limitation.

## Conclusion

Three themes shaping South Asian male survivors’ post-abuse journeys were identified to illustrate familial pressures around disclosure, taboos on sex and mental health, and barriers for criminal justice and mental health systems. Accounts provide a nuanced perspective on how shame, stigma, and secrecy is experienced by survivors, who navigated competing interests and relationships with often negative effects on their psychological wellbeing. Our findings illustrate traditional values and acculturations may obstruct acknowledgement and recovery for sexual violence victims in ethnic minority groups, who fear bringing shame and dishonour to their families. Barriers to access and distrust of ‘systems’ highlights the potential cultural dissonance in how support and justice resources are designed, amplifying the secrecy and unchallenged nature of sexual abuse perpetration in marginalized communities. This study gives a voice to a doubly disenfranchised group at the intersection of overlapping forms of stigma and violence. With sensitivity to the cultural factors elucidated here, researchers and providers can develop better outreach, treatment, and policies to assist South Asian male survivors along their journey to recovery.

## Electronic supplementary material

Below is the link to the electronic supplementary material.

Supplementary Material 1

## Data Availability

The datasets generated and/or analyzed during the current research are not publicly available as individual privacy could be compromised but are available from the corresponding author on reasonable request.
