# Supplementary material for: ‘We don’t talk about that around here’: an interpretative phenomenological analysis (IPA) of South Asian male survivors’ experiences of childhood sexual abuse in the UK
Source: BMC Psychol. 2025 Aug 13;13:910. doi: 10.1186/s40359-025-02706-z (PMC12345098; doi:10.1186/s40359-025-02706-z)
Supplement: Supplementary file 1 — Supplementary Material 1 [file 40359_2025_2706_MOESM1_ESM.docx]

**Schedule for semi-structured interviews with example questions**

| **Section** | **Example Questions** |
| --- | --- |
| **Initial Rapport Building** | So (name), how old are you? What are your current circumstances (e.g., job, family as an if  appropriate – survivors to advise). |
| **Free recall about experiences** | I would like to ask whether you can tell me anything you’d like to about your experience as a male survivor. If you’d prefer not to take this approach, don’t worry, I have questions we can start with instead |
| **Ethnicity** | In the questionnaire we sent before the interview you mentioned that you identify as […]: can you tell me a little more about your background?  Can you describe your experience as someone who identifies as a […] man who has survived sexual violence? |
| **Disclosure** | Did you ever disclose your experiences to someone? Who did you disclose to? Describe your experience of telling others about your experiences of sexual violence? How did you decide?  **If participant has not disclosed [if appropriate]**  Could you tell me anything about why you have not disclosed before today?  Could you tell me more about what motivated you to take part in this interview and speak about your experiences? |
| **INTERVIEWER TO CONDUCT A WELLBEING CHECK & OFFER A COMFORT BREAK** | |
| **Support** | Could you tell me more about how you managed your wellbeing after the experience?  Did you ever feel the need to access support?  Describe your experience of accessing a support service? How did you decide to seek support? What was your experience of the  service?  **If participant did not seek support in OR has never engaged with some form of support**  **attempt to explore this [if appropriate]** |
| **Reporting, Police, and the Criminal Justice System** | Could you tell me about your experience with reporting to the police? What motivated you to report to the police?  **OR**  Could you tell me more about your decision to NOT report? If you cast back your mind back, describe what influenced that decision? |
| **Conclusion** | What would you say to support services and policy makers about your experiences? Is there anything you would like to see happening in our society?  What advice would you give to another survivor from your ethnicity?  Is there anything else you would like to add? |
| **INTERVIEWER TO PAUSE AND STOP RECORDING, CONDUCT WELLBEING CHECK, & DEBRIEF PARTICIPANTS** | |
